# Stress-Based Production, and Characterization of Glutathione Peroxidase and Glutathione S-Transferase Enzymes From *Lactobacillus plantarum*

**DOI:** 10.3389/fbioe.2020.00078

**Published:** 2020-02-27

**Authors:** Lamiaa A. Al-Madboly, Safaa M. Ali, Esmail M. El Fakharany, Amany E. Ragab, Eman G. Khedr, Khaled M. Elokely

**Affiliations:** ^1^Department of Pharmaceutical Microbiology, Faculty of Pharmacy, Tanta University, Tanta, Egypt; ^2^Nucleic Acid Research Department, Genetic Engineering and Biotechnology Research Institute (GEBRI), City for Scientific Research and Technology Applications, Alexandria, Egypt; ^3^Protein Research Department, Genetic Engineering and Biotechnology Research Institute GEBRI, City for Scientific Research and Technology Applications, New Borg EL Arab, Egypt; ^4^Department of Pharmacognosy, Faculty of Pharmacy, Tanta University, Tanta, Egypt; ^5^Department of Biochemistry, Faculty of Pharmacy, Tanta University, Tanta, Egypt; ^6^Department of Pharmaceutical Chemistry, Faculty of Pharmacy, Tanta University, Tanta, Egypt; ^7^Institute for Computational Molecular Science, and Department of Chemistry, Temple University, Philadelphia, PA, United States; ^8^Division of Pharmaceutical Sciences, Arnold and Marie Schwartz College of Pharmacy and Health Sciences, Long Island University, Brooklyn, NY, United States

**Keywords:** *Lactobacillus plantarum*, glutathione s-transferase, glutathione-peroxidase, enzymatic activity, purification

## Abstract

More attention has been recently directed toward glutathione peroxidase and s-transferase enzymes because of the great importance they hold with respect to their applications in the pharmaceutical field. This work was conducted to optimize the production and characterize glutathione peroxidase and glutathione s-transferase produced by *Lactobacillus plantarum* KU720558 using Plackett-Burman and Box-Behnken statistical designs. To assess the impact of the culture conditions on the microbial production of the enzymes, colorimetric methods were used. Following data analysis, the optimum conditions that enhanced the s-transferase yield were the De Man-Rogosa-Sharp (MRS) broth as a basal medium supplemented with 0.1% urea, 0.075% H_2_O_2_, 0.5% 1-butanol, 0.0125% amino acids, and 0.05% SDS at pH 6.0 and anaerobically incubated for 24 h at 40°C. The optimum s-transferase specific activity was 1789.5 U/mg of protein, which was ~12 times the activity of the basal medium. For peroxidase, the best medium composition was 0.17% urea, 0.025% bile salt, 7.5% Na Cl, 0.05% H_2_O_2_, 0.05% SDS, and 2% ethanol added to the MRS broth at pH 6.0 and anaerobically incubated for 24 h at 40°C. Furthermore, the optimum peroxidase specific activity was 612.5 U/mg of protein, indicating that its activity was 22 times higher than the activity recorded in the basal medium. After SDS-PAGE analysis, GST and GPx showed a single protein band of 25 and 18 kDa, respectively. They were able to retain their activities at an optimal temperature of 40°C for an hour and pH range 4–7. The 3D model of both enzymes was constructed showing helical structures, sheet and loops. Protein cavities were also detected to define druggable sites. GST model had two large pockets; 185Å3 and 71 Å3 with druggability score 0.5–0.8. For GPx, the pockets were relatively smaller, 71 Å3 and 32 Å3 with druggability score (0.65–0.66). Therefore, the present study showed that the consortium components as well as the stress-based conditions used could express both enzymes with enhanced productivity, recommending their application based on the obtained results.

## Introduction

The use of enzyme technologies in the pharmaceutical research and industrial production fields is growing day by day. Using enzymes as medications have two important advantages that discriminate them from other kinds of drugs. First, enzymes often have strong affinity and specificity to their targets. Second, enzymes act as catalyst, which convert the target molecules into the required products. These two advantages make enzymes more potent and specific medicaments that can exert superior therapeutic effect within the body than smaller molecules. Based on these properties, many enzyme drugs have been developed for treatment of a wide range of diseases in which oxidative stress is involved such as several age-related conditions (i.e., cardiovascular diseases, chronic obstructive pulmonary disease, chronic kidney disease, neurodegenerative diseases, and cancer Vellard, 2003; McNeil et al., 2016; Liguori et al., 2018. Glutathione peroxidase (GPx) was used as potent antioxidant as it could decompose H_2_O_2_ to H_2_O protecting the biological molecules damage, inactivation, cross-linking and fragmentation, and peroxidation. The decrease of GPx activity is associated with the increase in the movement of hydrogen peroxide leading to activation of nuclear factor kappa β-related inflammatory pathways and direct damage of the tissue (Yu and Chung, 2006). Interestingly, Zhang et al. (2000) reported that oral administration of glutathione peroxidase-mimetic (bxt-51072) showed a potent anti-oxidant effect protecting the inflamed colonic mucosa from nitration and oxidation in patients with ulcerative colitis.

Glutathione s-transferases (GSTs) are important family of enzymes which play a great role in the binding, transformation, and detoxification of a variety of exogenous and endogenous electrophils (Oakley, 2011). GSTs are able to bind to hydrophobic non-substrates in a process termed “ligandin” which is usually used for storage, sequestration and drug transportation (Skopelitou et al., 2012). Apostolopoulos et al. (1993) succeeded to produce monoclonal antibodies against breast cancer using a glutathione-s-transferase-MUC1 bacterial fusion protein providing a useful diagnostic or therapeutic agent for breast cancer. Another example is the use of fusion proteins with multiple distinct enzymatic activities through engineering. A recombinant chimeric trifunctional enzyme with glutathione peroxidase, superoxide dismutase, and glutathione-s-transferase activities was generated (Yan et al., 2008). This enzyme was able to scavenge reactive oxygen species, providing several applications in medicine and environmental fields. The examples mentioned above highlight the potential use of engineered proteins and bacterial strains in the biotechnological and medical fields.

Glutathione peroxidase and s-transferase were known to present in yeasts, protozoa, metazoa, fungi, and bacteria (Arca et al., 1990; Tamaki et al., 1990; Datta et al., 1994; Jung et al., 1996; Yan et al., 2008; Zotta et al., 2017). To best of our knowledge, no previously published data were found about the optimization of medium components to improve the production of such enzymes from *L. plantarum*. Statistical methods used for enhancing the production of glutathione s-transferase and peroxidase enzymes has the advantage of screening many variables affecting the production through Placket-Burman design. To study three levels (−1, 0, +1) for each significant independent variable, Box-Behnken design is used to produce enzymes in maximal titers. Hence, experimental designs are considered valuable approaches for optimizing the production of different bio-products and they were highlighted in the literature (Bezerra et al., 2008; Ali et al., 2013).

Enzymes obtained from animal or plant sources were unable to meet the current requirements, converting the attention of the researchers to the microbial sources, which are characterized by broad biochemical diversity. Therefore, microorganisms became a preferable source of these enzymes due to the rapid growth rates, limited cultivation space, and the ease of their genetic manipulation that might lead to the production of enzymes with desirable biotechnological applications (Rajkumar et al., 2013). Furthermore, Allocati et al. (2009) reported that bacterial GSTs and GPxs, which were highly stable and characterized by the catalytic activity of a variety of reactions, undoubtedly, represent an effective resource for the future. Additionally, the potency to produce both enzymes extensively differs among the strains of lactic acid bacteria and is significantly affected by the medium composition and culture conditions. Hence, it is essential to optimize such conditions through suitable experimental designs, which is common in biotechnology, to enhance productivity (Zareian et al., 2013). In this context, the current work aimed to optimize the production of GPx as well as GST by *L. plantarum* KU720558 using the Placket-Burman and Box-Behnken experimental designs. Additionally, purification, characterization and sequencing would be conducted due to the importance of such enzymes in the pharmaceutical and medical fields.

## Materials and Methods

### Bacterial Strain and Culture Conditions

*Lactobacillus plantarum* KU720558 strain was used for optimizing the production of glutathione s-transferase and -peroxidase. It was previously isolated, identified and tested for reduced glutathione production in the study of Al-Madboly et al. (2017). This strain is producer to candidate enzymes involved in glutathione synthesis and maintenance, and hence selected for the current investigation. The test strain is routinely grown in De Man-Rogosa-Sharp (MRS) broth and anaerobically incubated for 24 h at 37°C. The medium is prepared by adding a specific amount of the following components to 1 L of distilled water at 60°C. The components include; peptone 10 g, Lab-Lemco powder 8 g, yeast extract 4 g, glucose 20 g, sorbitan mono-oleate 1 ml, dipotassium hydrogen phosphate 2 g, sodium acetate 3H_2_O 5 g, triammonium citrate 2 g, magnesium sulfate 7H2O 0.2 g, manganese sulfate 4H_2_O 0.05 g, and finally adjusted pH 6.2 ± 0.2 at 25°C. The added components were mixed till complete dissolution, dispensed into appropriate containers and sterilized by autoclaving at 121°C for 15 min. All components were purchased from OXOID (UK).

Stock culture was preserved in MRS broth containing 20% (v/v) glycerol at – 20°C. The strain was activated in MRS broth at 37°C for 24 h then subcultured for two consecutive passages in MRS broth at 37°C for 24 h before use.

### Growth Profile and the Enzymatic Activity

*L. plantarum* KU720558 strain was grown in MRS broth, as a basal medium, at 37°C under anaerobic conditions. The optical density (OD) of the bacterial cells was measured at 660 nm spectrophotometrically at 0, 2, 4, 6, 8, 10, 24, 48, and 72 h using a disposable cuvette and hence growth curve can be drawn. Each measurement represented the mean of three repeated experiments (Zhang et al., 2007; Olson and Aryana, 2012). For each time interval, the enzymatic activities were determined in the cell lysate as follows.

### Preparation of the Cell Lysate

It was prepared as previously reported by Zhang et al. (2007) and Al-Madboly et al. (2017). Briefly, overnight cultures grown on MRS agar at 37°C were used to inoculate flasks containing MRS broth and were incubated under anaerobic conditions until log phase. Bacteria were harvested by centrifugation (5,000 × g) at 4°C for 15 min. Next, the pellet was washed twice with phosphate buffered saline (PBS, pH 7.4) and then re-suspended in PBS. The cell suspension was disrupted using an ultrasonicator (Branson Sonic Power, USA) in an ice bath. Cell debris was removed by centrifugation (10,000 × g for 10 min at 4°C). Protein content was estimated in the cell lysate using protein assay kit (Bio-Rad, USA) and this was conducted according to the manufacturer's instructions.

### Determination of Glutathione S-Transferase (GST) Activity

The intracellular GST activity was measured through a GST assay kit (Biodiagnostics, Egypt) based on the colorimetric method described by Habig et al. (1974). This reaction is dependent on quantifying the engagement of 1-chloro-2,4-dinitrobenzene with the reduced glutathione at 340 nm. The increase in the absorbance is directly proportional to the rate of GST activity in the sample. GST positive control was supplied within the assay kit. In addition, a negative control of bovine serum albumin was also assayed. Single unit of GST activity is equivalent to the amount of enzyme giving 1 μmol of GS-DNB conjugate/min under the assay conditions. GST specific activity was expressed as (U/mg protein). Each measurement represented the mean of three repeated experiments. For each stage, at least two independent samples were assayed, and each sample was assayed at least twice, in duplicate each time.

### Determination of Glutathione-Peroxidase (GPx) Activity

GPx activity was determined through a GPx assay kit (biodiagnostics, Egypt) based on the colorimetric method described by Paglia and Valentine (1967). The principle of assay depends on the indirect measurement of the GPx activity. To assay GPx activity, cell lysate was transferred to a solution containing GR, GSH, and NADPH. Initiation of the enzymatic reaction was achieved by adding the substrate, hydrogen peroxide and the absorbance was recorded. The reduction rate in the absorbance (A_340_) is directly proportional to the GPx activity in the sample. Bovine serum albumin was used in the reaction mixture as a negative control. One unit of GPx activity was defined as the amount of enzyme required to oxidize 1 nmol of NADPH per min under the above-described assay conditions. The specific enzyme activity was expressed as units per mg of protein. For each measurement, three repeated experiments were carried out to calculate the mean value. For each stage, at least two independent samples were assayed, and each sample was assayed at least twice, in duplicate each time.

### Optimization of GST and Gpx Production

#### Plackett-Burman Screening Design and Statistical Analysis of Data

The experimental design of Plackett and Burman (1946) was used to identify and screen the influencing parameters in the medium components affecting the production of the GST and GPx enzymes. Thirteen test variables were evaluated at two levels, −1 for the low and +1 for the high, depending on a Plackett-Burman matrix design (Table 1). In the current study, 16 trials were included in a design matrix to study the selected 13 parameters (Ali et al., 2013). Plackett-Burman experimental design was dependent on a first-order model equation: Y = ß_0_ +∑ ßiXi, where Y was the response (GST or GPx activity), ß_0_ was the model intercept, ßi was the linear coefficient, and Xi was the level of the independent parameter. This model was used to determine the significant variables affecting the production of the enzymes.

**Table 1:** Test parameters and levels used in Plackett-Burman design in order to screen for culture conditions affecting the enzymes production.

**Plackett-Burman design matrix**														**Enzyme specific activity (U/mg of protein)**	
**Trial no**.	**Na Cl (%)**	**Bile salt (%)**	**H_**2**_O_**2 **_(%)**	**SDS (%)**	**Ethanol (%)**	**Urea (%)**	**1-Butanol (%)**	**Aerobic incubation**	**Amino acids**	**pH**	**Temperature (°C)**	**Incubation time (h)**	**Cooling (h)**	**GST**	**GPx**
1	5	0.05	0	0.05	0	0	1	+	+	6	30	24	0	502 ± 0.04	14 ± 0.08
2	1	0.05	0.05	0	2	0.1	0	+	+	6	30	18	24	347 ± 0.07	5 ± 0.13
3	5	0	0.05	0.05	0	0	1	–	+	8	30	18	0	680 ± 0.03	19 ± 0.33
4	1	0.05	0	0.05	2	0.1	0	+	–	8	40	18	0	320 ± 0.10	244 ± 0.07
5	1	0	0.05	0	2	0	1	–	+	6	40	24	0	907 ± 0.03	19 ± 0.12
6	1	0	0	0.05	0	0.1	0	+	–	6	40	24	24	54 ± 0.23	21 ± 0.11
7	5	0	0	0	2	0.1	1	–	+	8	30	24	24	89 ± 0.14	73 ± 0.13
8	5	0.05	0	0	0	0	1	+	–	6	40	18	24	240 ± 0.08	7 ± 0.04
9	5	0.05	0.05	0	0	0.1	0	+	+	8	30	24	0	181 ± 0.12	39 ± 0.04
10	1	0.05	0.05	0.05	0	0	1	–	+	6	40	18	24	620 ± 0.05	27 ± 0.04
11	5	0	0.05	0.05	2	0	0	+	–	8	30	24	0	154 ± 0.22	117 ± 0.05
12	1	0.05	0	0.05	2	0	0	–	+	8	40	18	24	86 ± 0.07	18 ± 0.021
13	5	0	0.05	0	2	0.1	0	–	–	6	40	24	0	588 ± 0.05	454 ± 0.01
14	1	0.05	0	0.05	0	0.1	1	–	–	8	30	24	24	26 ± 0.13	119 ± 0.01
15	5	0	0.05	0	2	0.1	1	+	–	6	40	18	24	148 ± 0.11	54 ± 0.014
16	1	0	0	0	0	0	0	–	–	8	30	18	0	89 ± 0.05	5 ± 0.34

+, *Presence of variable*.

–, *Absence of variables*.

±, *for standard deviation*.

Optimization medium was prepared as previously described by Al-Madboly et al. (2017). The concentrations of the components in each trial were prepared according to Table 1. The resulted GST and GPx data were statistically analyzed where Experimental Design software was used for data analysis and the coefficients were determined (Steppan et al., 1999).

#### Box-Behnken Experimental Design and Statistical Analysis of Data

The optimum conditions for GST and GPx production were described using design (Box and Behnken, 1960). Separate matrices were designed for the test enzymes and each matrix consisted of 13 trials to evaluate the most significant factors affecting the production as shown in Table 2.

**Table 2:** Box-Behnken design matrices along with the experimental and predicted values of s-transferase and peroxidase specific activities.

Box-Behnken design matrices^*^				**Experimental and predicted enzyme specific activity (U/mg protein)**			
**Trail no**.	**X1**	**X2**	**X3**	**Experimental GST**	**Predicted GST**	**Experimental GPx**	**Predicted GPx**
1	0	−1	−1	500 ± 0.11	486.75	460 ± 0.05	392.75
2	0	1	−1	1211 ± 0.08	1195.25	119 ± 0.11	213
3	0	−1	1	1372 ± 0.02	1387.75	640 ± 0.08	546
4	0	1	1	695 ± 0.33	708.25	390 ± 0.13	457.25
5	−1	−1	0	1390 ± 0.07	1431.75	11 ± 0.15	77.375
6	−1	1	0	1532 ± 0.02	1576.25	49 ± 0.22	45.875
7	1	−1	0	898 ± 0.13	853.75	132 ± 0.13	226.875
8	1	1	0	780 ± 0.15	738.25	148 ± 0.17	81.625
9	−1	0	−1	1400 ± 0.04	1371.5	172 ± 0.22	172.875
10	−1	0	1	1860 ± 0.03	1802.5	101 ± 0.22	128.625
11	1	0	−1	830 ± 0.14	887.5	96 ± 0.14	68.375
12	1	0	1	842 ± 0.22	870.5	511 ± 0.08	510.125
13	0	0	0	890 ± 0.18	890	182 ± 0.13	182

* *For s-transferase; X1, 1-butanol; X2, H_2_O_2_; X3, amino acids. For peroxidase; X1, urea; X2, bile salt; X3, Na Cl*.

Each significant parameter was tested in three levels (−1, 0, +1) for (low, middle, and high values, respectively) and details were presented in Table S1. To study the interactions between test variables, Box-Behnken experimental design was performed. The variables; X1, X2, and X3 differed from enzyme to another; they represented amino acids, H_2_O_2_ and 1-butanol for s-transferase while urea, bile salt, and sodium chloride in case of peroxidase, respectively. A second order polynomial function could predict the optimal point for each enzyme separately and hence assess the quality of the design. The polynomial model equation for each enzyme was expressed by a coefficient of determination (*R*^2^). Multiple linear regression (*t*-values, *P*-values, and confidence levels) estimated.

#### Purification and Characterization of GST and GPx Enzymes

Purification of GST and GPx from the cell lysate was conducted using column chromatography according to Cha et al. (2001) and Yamashita et al. (2012). For GST purification, it was carried out using an FPLC system. Briefly, the cell extract was loaded onto a Q-Sepharose ion exchange column (2.6 × 12 cm) which was previously equilibrated with 50 mM sodium phosphate buffer (pH 7.4). GST was eluted from the column using a linear gradient of 0–0.6 M Na Cl. The active fractions were pooled and subjected to GSH-agarose affinity chromatography. The sample was loaded on the column and then washed with the same phosphate buffer containing 0.1 M NaCl. GST was eluted with 50 mM Tris-HCl buffer (pH 9.5) containing 10 mM GSH Cha et al. (2001).

For GPx purification, the cell lysate was passed through a DEAE-Sephadex A-50 column (60 × 200 mm) equilibrated in 20 mM Tris-HCl buffer (pH 7.4) containing 1 mM EDTA-disodium and 1 mM GSH. Fractions were concentrated and dialyzed against the same buffer used in equilibration, then passed through another DEAE Sephadex-A50 column (60 × 300 mm). The column was washed with the buffer and the enzymes were eluted with a linear gradient of Na Cl (0–0.2 M). The active fraction was concentrated to 5 ml with an Amicon stirred cell 8050 equipped with a PM-50 membrane (Millipore, USA), run through a gel filtration chromatography Sephacryl S-200 column (16 × 600 mm), and eluted with 10 mM Tris-HCl buffer (pH 6.8) containing 100 mM Na Cl, 1 mM EDTA- disodium, and 1 mM GSH. Then the active fraction was concentrated with an Amicon stirred cell 8050, equilibrated with 20 mM HEPES buffer (pH 6.8) containing 1 mM EDTA-disodium and 1 mM GSH, purified with a Mono Q column (5 × 20 mm, Pharmacia), washed with buffer, and eluted with a linear gradient of 0–0.5 M Na Cl (Yamashita et al., 2012).

The concentrated purified GST and GPx solutions were subjected to quantification of the protein content as well as determination of the specific activity as previously determined by Zhang et al. (2007) and the enzymatic assay kit. The purified GST and GPx samples were subjected to Sodium Dodecyl Sulfate Polyacrylamide Gel Electrophoresis (12% SDS-PAGE) and isoelectric focusing (IEF) that were carried out according to Laemmli (1970) and Chee et al. (2014).

For characterization of the purified enzymes, GST and GPx solutions were divided into a number of aliquots to investigate the effect of temperature and pH on the enzymatic properties. The test enzymes were pre-incubated in Tris-HCl (pH 7.0) at different temperature degrees ranged between (4–70°C) for 15, 30, 45, and 60 min. Following pre-incubation at higher temperatures, the enzymes were immersed in an ice bath then the residual intact activity was measured through an assay kit (Biodiagnostics, Egypt). In addition, the enzymatic stability was also assessed as a function of pH ranging from 4 to 10 for 60 min at room temperature using different buffer solutions. Additionally, the enzymatic activity was also evaluated in the presence of different metal ions at pH 7 (50 mM Tris-HCl) for 60 min at room temperature. The residual activity in the treated and control samples were plotted against the time of exposure for treatment as relative activity. The control samples were considered as 100% activity (Shi et al., 2014; Wang et al., 2017). Each measurement represented the mean of three repeated experiments.

#### Amino Acids Sequencing

The amino acid residues forming the enzymatic structures were sequenced using a protein sequencer (Automated Edman Protein Seq. Hewlett Packard G1000A with an on-line PTH-amino acid analyzer based on the HP 1090M HPLC). Searching for homology, multiple sequence alignment and phylogenetic tree construction was done using NCBI BLASTp tool. (https://blast.ncbi.nlm.nih.gov/Blast.cgi?PROGRAM=blastp&PAGE_TYPE=BlastSearch&LINK_LOC=blasthome). EMBL database link for GPx (C0HLL8); https://www.uniprot.org/uniprot/C0HLL8. For GST (C0HLL7) link; https://www.uniprot.org/uniprot/C0HLL7#names_and_taxonomy. The predicted pIs of GST and GPx sequences was performed using Compute pI/MW tool:EXPASY (http://web.expasy.org/compute_pi). Clustal Omega was also used to construct multiple sequence alignements (https://www.ebi.ac.uk/Tools/msa/clustalomega).

#### Building the 3D Model of Sequences

Prime was used to build the 3D model of both sequences (Jacobson et al., 2002, 2004; Schrödinger, 2018a). The non-redundant NCBI database was selected to identify the highest homologous protein crystal structures using BLAST homology search engine. The templates and queries were aligned using ClustalW and the alignment scores were computed based on BLOSUM62 similarity matrix. The default penalty values for gap opening and extension were defined. Rotamers for conserved residues were retained. Side chains were optimized and non-template residues were subjected to rigorous minimization. Loops were then optimized using serial loop sampling allowing for a minimum distance of 0.7 Å for vdW radii. The models were then validated using BioLuminate (Beard et al., 2013; Schrödinger, 2018b).

## Results

### Growth Profile and the Enzymatic Activity

**Figure 1:**
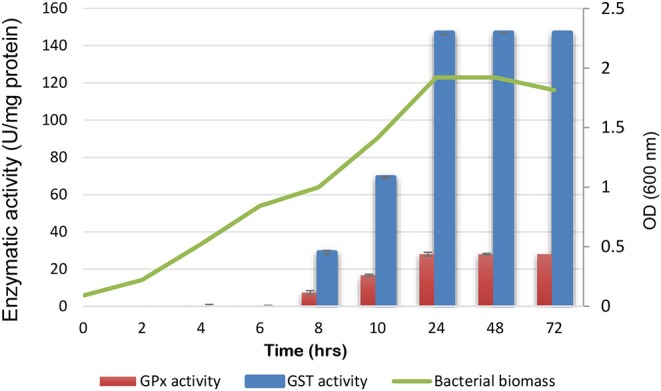
Time-course of glutathione peroxidase and s-transferase production by *L. plantarum* along with the biomass.

The time-course of the enzymatic activities along with the biomass measurements of the test organism; *L. plantarum* KU720558 strain were shown in Figure 1. The highest biomass (1.92) was measured at 24 h of incubation. Maximal GST and GPx activities (147 and 28 U/mg protein) were measured at the end of the logarithmic growth phase, which were remained constant until the end of the growth cycle.

### Optimization of GST and Gpx Production

#### Screening of Significant Factors by Plackett-Burman Statistical Design

**Figure 2:**
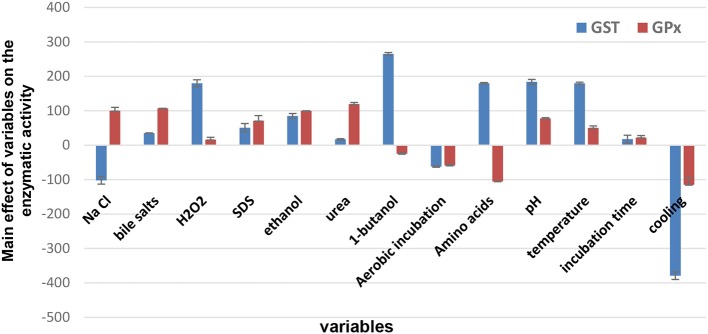
The main effects of culture conditions on glutathione-s-transfrase and glutathione peroxidase enzymes produced by *L. plantarum* KU720558 based on Plackett-Burman design results. It was shown that 0.05% H2O2, 0.05% SDS, 0.1% urea, 1% 1-butanol, 0.05% bile salt, 2% ethanol, 0.05% equally mixed amino acids, pH 6 and anaerobic incubation at 40°C for 24 h enhanced GST productivity. For GPx, Na Cl (5%), bile salt (0.05%), H2O2 (0.05%), SDS (0.05%), ethanol (2%), pH 6, urea (0.1%), and anaerobic incubation at 40°C for 24 h increased the productivity.

Concerning the Placket-Burman results, the amounts of GST and GPx produced in each trial were expressed in terms of activity. For GST, the activity ranged between 26–907 U/mg of protein with emphasis on trial number 5, which showed the highest activity, as recorded in Table 1. It was found that 0.05% H_2_O_2_, 0.05% SDS, 0.1% urea, 1% 1-butanol, 0.05% bile salt, 2% ethanol, 0.05% equally mixed amino acids, pH 6 and anaerobic incubation at 40°C for 24 h enhanced GST productivity, as observed from Figure 1. Among these, the most significant variables were H_2_O_2_, 1-butanol and amino acids. Placket-Burman results for GPx revealed variation in the activity that ranged between 5 and 454 U/mg of protein, focusing on trial number 13, which recorded the highest activity (Table 1). Sodium chloride (5%), bile salt (0.05%), H_2_O_2_ (0.05%), SDS (0.05%), ethanol (2%), pH 6, urea (0.1%), and anaerobic incubation at 40°C for 24 h stimulated GPx productivity (Figure 2). The top three significant factors were sodium chloride, bile salt, and urea.

#### Regression Analysis of Placket-Burman Design

The regression analysis of Placket-Burman design was conducted for fitting the mathematical model to the experimental results to obtain an optimal response, for more details see Table S2. Analysis of variance (ANOVA) was used to estimate the significance of the model coefficient. The *p*-values represented the significance of each coefficient, Variables with confidence levels {(1 – *p-*value)^*^100} >61.69% were considered significant for s-transferase, whereas those with confidence levels >86.12% were considered significant for peroxidase. The models were significant (*p* < 0.05), and the values of the coefficient determination *R*^2^ were 0.894 and 0.962 for GST and GPx, respectively, indicating that 89.4 and 96.2% of the variability in response could be explained by the models. The quality of the fitting model equations for the yields of the enzymes could be presented as follows:

Y_s−transferase_ = 314.4375 + 87.15024x_3_ + 125.5463x_7_ + 88.84293x_9_+ 84.41432x_11_

Y_peroxidase_ = 77.1875 + 50.34368x_1_+ 53.22154x_2_ + 58.08034x_6_.

### Optimization of the Screened Significant Factors Using Box-Behnken Design

The Box-Behnken design determined three levels for each significant parameter to obtain optimal GST and GPx production, as recorded in Table 2. The maximum experimental values for GST and GPx production were 1,860 and 511 U/mg of protein, respectively, while the predicted responses were 1802.5 and 510.125 U/mg of protein, respectively. The close correlation between the experimental and predicted data reflected the appropriateness of the model.

### Developing a Regression Model

Regression analysis of Box-Behnken design was performed to fit the response function (enzyme activity) with the experimental data, for more detailed information, see Table S3. The analysis of variance for the significant variables indicated that enzyme activity could be well-described by a polynomial model with a coefficient of determination (*R*^2^ = 0.991 and 0.884 for GST and GPx, respectively). It was also noted that *R*^2^ for GST and GPx was in close agreement with the adjusted *R*^2^ (0.989 and 0.881, respectively). The closeness of the *R*^2^ values to 1.0 reflected the strength of the model and predicted the response better. The *F*-values of the model were 25.43371 (for GPx) and 37.56945 (for GST), and the *P* > *F* (<0.023898 and 0.006283, respectively), indicating that the terms of the models were significant (Table 3) and that the models could accurately represent the experimental data.

**Table 3:** ANOVA of fit model for GST and GPx activities.

		**df**	**SS**	**MS**	**F**	**Significance F**
GPx activity	Regression	9	1858166	206462.9	37.56945	0.006283
	Residual	3	16486.5	5495.5		
	Total	12	1874653			
GST activity	Regression	9	420104.3	46678.26	25.43371	0.023898
	Residual	3	55058.75	18352.92		
	Total	12	475163.1			

Significant interaction was determined at *p* < 0.05, and the models can be expressed as follows:

Y_s−transferase_ = 890 − 354X_1_ + 7.25X_2_ + 103.5X_3_ + 274.25${\text{X}}_{1}^{2}$ − 14.25${\text{X}}_{2}^{2}\;+\;$68.75${\text{X}}_{3}^{2}$ − 65X_1_X_2_ − 112X_1_X_3_ − 347X_2_X_3_

Y_peroxidase_ = 181 + 69.25X_1_ − 67.125X_2_ + 99.375X_3_ − 139.625${\text{X}}_{1}^{2}\;+\;$42.625${\text{X}}_{2}^{2}\;+\;$177.625${\text{X}}_{3}^{2}$ − 5.5X_1_X_2_ + 121.5X_1_X_3_ + 22.75X_2_X_3_.

### Validating the Optimum Concentrations of the Factors

After data analysis, the optimized medium for GST consisted of MRS broth to which H_2_O_2_ (0.075%), SDS (0.05%), amino acids (0.0125%), 1-butanol (0.5%), and urea (0.1%) were added. The pH of the medium was adjusted to 6 and incubated for 24 h at 40°C under anaerobic conditions. A verification experiment was conducted to determine the fitness of the model using the optimal conditions, which resulted in an experimental GST of 1798.5 U/mg of protein that was similar to the predicted response of 1776.5 U/mg of protein, indicating 12 times the activity produced using the basal medium. For GPx, the optimum medium was composed of MRS broth supplemented with 0.170227% urea, 0.025% bile salt, 7.5% Na Cl, 0.05% H_2_O_2_, 0.05% SDS, and 2% ethanol, at pH 6.0, and anaerobically incubated at 40°C for 24 h. Validation of this model was also performed by conducting the experiment under these conditions. This validation resulted in an experimental GPx activity of 612.5 U/mg of protein, which was close to the predicted activity (614.9 U/mg of protein), showing a 22-fold increase in the productivity compared to the control.

### Purification of GST and GPx Enzymes

**Figure 3:**
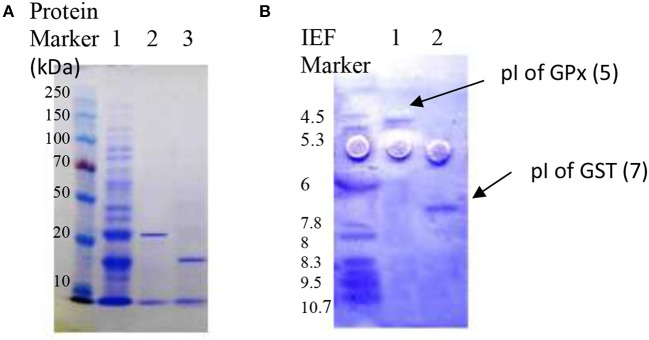
**(A)** SDS-PAGE of GSTs and GPx proteins from *Lactobacillus plantarum* showing the total proteins of the cell lysate before purification (lane 1), the purified GST with a single band at 25 kDa (lane 2), and the purified GPx at 18 kDa (lane 3). The first lane to the left shows the protein marker (Biorad, Milan, Italy). **(B)** Isoelectric-focusing points of 7 μg of the purified GPx (lane 1), GSTs (lane 2), and the first lane to the left presents SERVA IEF marker.

The solutions of GST and GPx were purified using column chromatography. Table 4 summarized the overall purification procedure of GPx, which passed through different stages of gel filtration chromatography using DEAE-Sephadex A50, Sephacryl S-200, MonoQ ion exchange column resulting in 3-fold purified yield of 42.4%. For GST, it was purified 2-fold with 39.8% overall yield as recorded in Table 5. Additionally, the purified GST and GPx showed a single protein band weighing 25 and 18 kDa, respectively, following SDS-PAGE (Figure 3). The results of the isoelectric focusing experiment presented pIs of 7 and 5 for GST and GPx, respectively.

**Table 4:** Purification scheme of GPx from the cell lysate of *L. plantarum*.

	**Total protein in mg**	**Total activity in U**	**Specific activity U/mg**	**Yield (%)**	**Purification *n*-fold**
1-Crude extract	2.01	1231.1	612.5	100	–
2-DEAE-Sephadex A-50	1.73	1164	672.8	94.5	1.1
3-DEAE-Sephadex A-50	1.42	1110	781.7	90.1	1.28
4-Sephacryl S-200	1.16	1023.2	882.1	83.1	1.44
5-DEAE-Sephadex A-50	0.79	978.3	1238.4	79.5	2
6-Sephacryl S-200	0.47	917	1951.1	74.5	3.19
7-Mono Q	0.25	522	2088	42.4	3.41

**Table 5:** Purification scheme of GST from the cell lysate of *L. plantarum*.

	**Total protein in mg**	**Total activity in U**	**Specific activity U/mg**	**Yield (%)**	**Purification *n*-fold**
1-Crude extract	2.01	3596.9	1789.5	100	−
2- Q Sepharose	1.6	2867.2	1792	79.7	1
3- GSH agarose	0.36	1433	3980.6	39.8	2.22

### Characterization of GST and GPx Enzymes

**Figure 4:**
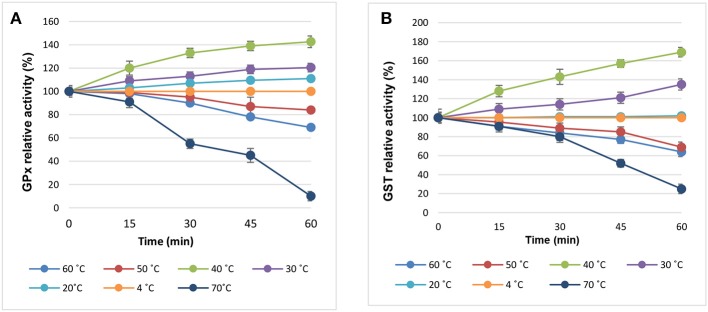
The biochemical properties of **(A)** GST and **(B)** GPx enzymes at different temperature degrees.

**Figure 5:**
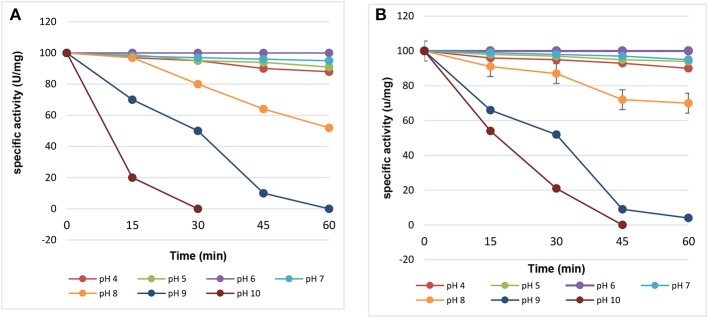
The biochemical properties of **(A)** GST and **(B)** GPx enzymes at different pH values.

Figure 4 presented the effect of temperature on the enzymatic activities. The results revealed improved activities when the enzymes were incubated at 30 and 40°C. Although the optimal activity for both enzymes was recorded at 40°C, the enzymes retained 70% of their activities at 50°C and lost about 36% of the activity at 60°C (Figure 4). A marked decrease in the relative activities of both enzymes (25%, GST; 10%, GPx) was noticed at 70°C. Furthermore, the effects of different pH values on the enzymatic activities were shown in Figure 5. It was found that GST and GPx were able to retain their activities at pH range 4–7 with optimal activity at pH 6. Slight reduction in the activity was recorded at pH 8. However, dramatic decrease in the enzymatic activity was noticed at high alkaline pH values after 60 min (Figure 4).

The results recorded in Table 6 presented the impact of different metal ions as well as reagents on the GST and GPx activities. It was found that Fe^2+^ and DTT markedly reduced the GST relative activity by 60 and 68%, respectively. On the reverse, Mn^2+^ increased the GST activity slightly to 101%. In contrast to other investigated treatments, which had no marked effect on GPx relative activity, Cu^2+^ showed the strongest decreasing effect (42.5%) followed by SDS (59.6%) and DTT (61.4%). However, Fe^2+^, Mn^2+^, triton X-100, and EDTA had a slight enhancing effect on the enzymatic relative activity.

**Table 6:** Impact of different metal ions and reagents on the GST and GPx relative activities.

		**Relative activity (%)**	
**Reagent**	**Concentration**	**GST**	**GPx**
No addition	−	100	100
Ca2^+^	5 mM	100	90.3
Mg2^+^	5 mM	89.3	97.1
Cu2^+^	5 mM	98.7	42.5
Zn2^+^	5 mM	65.4	86
Fe2^+^	5 mM	40	102
Mn2^+^	5 mM	101	101.3
K^+^	5 mM	100	98.3
Na2^+^	1 M	100	99
Triton X-100	0.2%	100	102
Tween 80	0.2%	100	95
SDS	10 mM	61	59.6
EDTA	10 mM	90	101
DTT	10 mM	32	61.4

### Sequence Analysis

**Figure 6:**
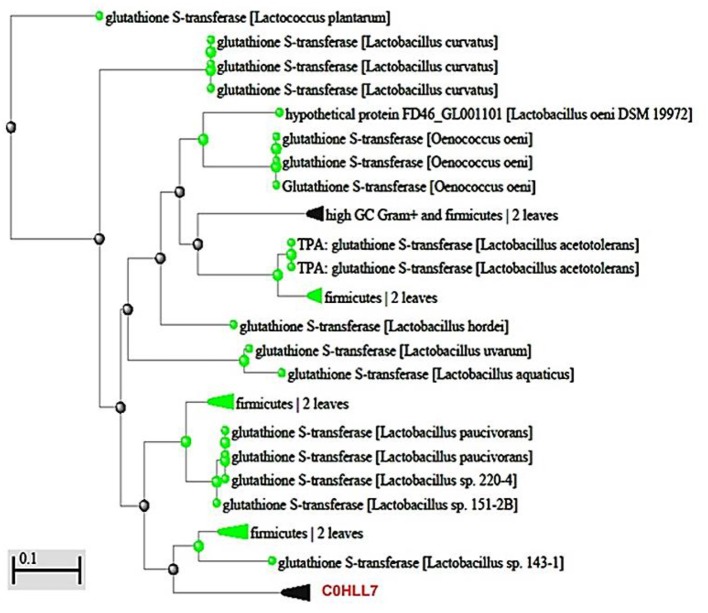
Phylogenetic relationship of GST (C0HLL7) amino acid sequence from *L. plantarum* with those of GST-producing bacteria. The scale indicates the evolutionary distance of 0.1 per site.

**Figure 7:**
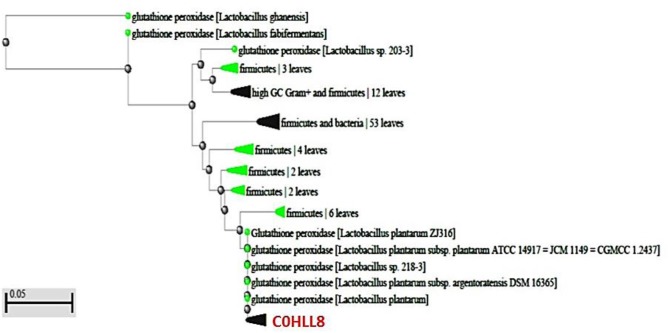
Evolutionary relationship between GPx (C0HLL8) amino acid sequence from *L. plantarum* and those of GPx-producing bacteria. The scale bar represents a distance of 0.05 substitutions per site.

In the present work, an amino acid sequences similarity check, done using BLASTp tool, confirmed that the two sequences were homologs of GST and GPx from *L. plantarum*. The closest relatives of GST amino acid sequence were hypothetical protein (GenBank KZU34095.1; *E*-value, 3e-24) and glutathione s-transferase from *L. plantarum* (GenBank OAZ73640.1; *E*-value, 2e-23) showing 100% identity and query cover sequence. For GPx, the closest sequences were two glutathione peroxidases from *L. plantarum* (GenBank WP_137638942.1; *E*-value, 7e-08) and (GenBank WP_024521195.1; *E*-value, 7e-08) presenting 76.67% identity and 96% query cover sequence. Additionally, both Query sequences of GST and GPx belonged to thioredoxin superfamily showing 73.3 and 76.6% identity, respectively, as confirmed by BLASTp tool (EMBL database; (http://www.ebi.ac.uk/Tools/msa/BLASTp/functional predection) (Figure S4). Furthermore, both sequences were deposited in the Uniprot databse under accession numbers C0HLL7 (Glutathione S-transferase) and C0HLL8 (Glutathione peroxidase) from *L. plantarum*. A phylogenetic tree was constructed for each enzyme to determine the evolutionary relationships of these enzymes among those in the database as shown in Figures 6, 7. Moreover, the predicted pIs of GST and GPx sequences performed using Compute pI/ tool: EXPASY was 6.93 and 5.22.

### Multiple Sequence Alignments

**Figure 8:**
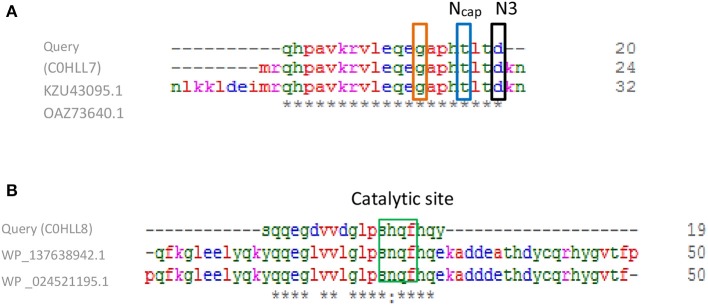
Multiple sequence alignments of **(A)** Query GST (C0HLL7) class Beta with *L. plantarum* GST (KZU43095.1) and in comparison with another *L. plantarum* GST (OAZ73640.1). The N-capping box residues shown in blue, N3 presented in black box and the glycine residue surrounded with red box. Conserved residues were presented as (*) which means identical residue and (:) means conserved substitution. **(B)** Query GPx (C0HLL8) with *L. plantarum* GPx (WP_137638942.1) and in comparison with another *L. plantarum* GPx (WP_024521195.1). Catalytic site was shown surrounded with green box. Multiple alignments were generated using CLUSTAL omega program with its default parameter setting (http://www.ebi.ac.uk/Tools/msa/clustalomega/).

Both query amino acid sequences were subjected to multiple sequence alignments using Clustal Omega with the top two protein sequences appeared in the hit list. For GST (C0HLL7), this alignment revealed 20 conserved residues. Two structural motifs were conserved in the GST sequence i.e., *N*-capping box (S/TXXD) as well as the hydrophobic motif, which were essential for the enzymatic folding and stability (Figure 8A). The *N*-capping box was consisted of Thr27 while the N3 region showed conserved Asp30. Interestingly, glycine residue was part of a buried local sequence: **G**-X-X-**h**-**T**-X-X-**D**. For Gpx multiple sequence alignments, Ser (S25) was conserved and present beside His (h26), Gln (q27), and f28. Moreover, His (h26) is semiconservative substitute to Asn (n26). This sequence was suggested to be the catalytic site as shown in Figure 8B.

### Construction of 3D Model

**Figure 9:**
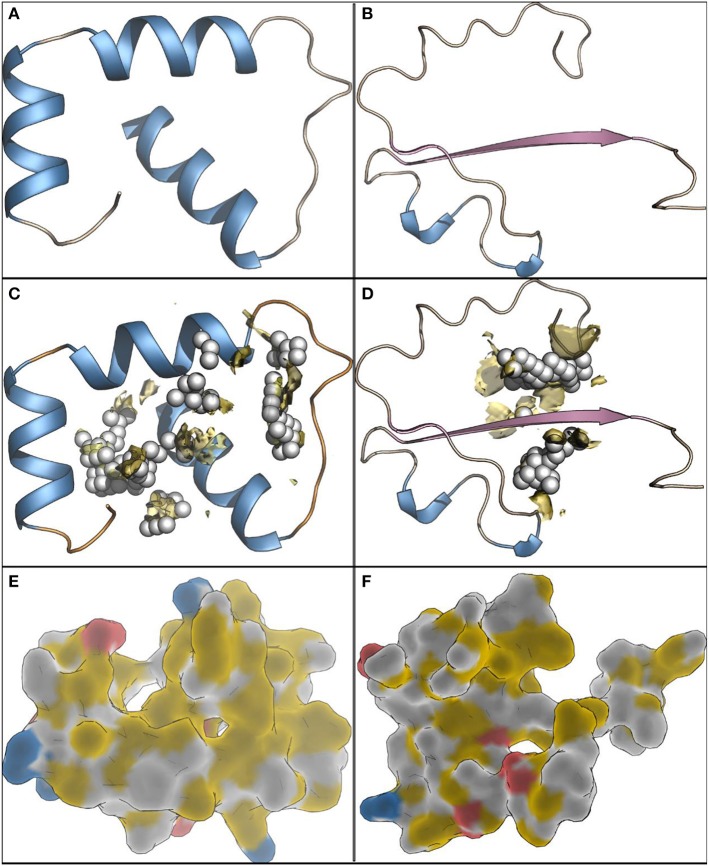
The 3D models of GST **(A)** and Gpx **(B)**, from *L. plantarum*, as cartoon. The helical structures are shown as blue, sheets as pink and loops thread like. The alpha carbons are shown as white spheres. The protein cavities **(C)** and **(D)** are shown as spheres and yellow surface. The highlight of the hydrophobicity and charges on protein surfaces **(E)** and **(F)**, yellow and white for hydrophobic regions, red for negatively charged surfaces and blue for the positively charged patches.

To construct the 3D model of GST (C0HLL7) sequence, the crystal structure of glutathione s-transferase enzyme (PDB ID: 4IQ1, https://www.rcsb.org) was selected as a template, which showed a Prime score of 61.0 and an expectation value of 0.31. The percent identity and similarity between the target sequence and selected template are 34 and 46%, respectively. The model was constructed with high confidence. The model shows helical structures and loops (Figure 9A).

The crystal structure of glutathione-dependent phospholipid peroxidase (PDB ID: 3CMI, https://www.rcsb.org) was selected as a template to build the 3D model of the GPx (C0LL8) sequence. The template showed a Prime score of 62.0 and an expectation value of 0.21. The percent identity between the template and the query is 35% and the similar amino acids are 50% of the sequence. This indicates the high validity of the constructed model. The generated model showed two helical structures, two strands and loops (Figure 9B). Possible protein cavities were explored to define druggable sites. GST model showed two considerably large pockets, 185 Å3 and 71 Å3 (Figure 9C), with druggability score in the range of 0.5–0.8. The two cavities are connected and can form a large pocket. While in case of the peroxidase model, it has two pockets as well with relatively smaller volumes, 71 Å3 and 32 Å3 (Figure 9D). The two pockets are connected and show druggability scores of 0.65 and 0.66. The two models were also mapped to identify the electrostatic properties of the surface patches (Figures 9E,F). Upon analyzing the structures of both enzymes, some thermal stability (up to 60°C and little at 70°C) appears to be due to a significant amount of hydrophobic regions on the protein surface while polar patches were smaller.

## Discussion

Carbon and nitrogen sources are crucial factors that should be selected accurately to develop an efficient production process. Rajkumar et al. (2013), reported different organic and inorganic nitrogenous sources that were tested, including peptone, beef extract, yeast extract, ammonium chloride, and sodium nitrate. They found that 1.5% (w/v) yeast extract enhanced the production of peroxidase (45.6 U/mg) from *Bacillus* sp. Allocati et al. (2009) reported that *Proteus miabilis* grown in the presence of 40 mM of KNO_3_ resulted in high GST levels. Iizuka et al. (1989) mentioned that the fermentation medium components of GST-producing *E. coli* included 1% glucose, 0.1% yeast extract, 1% peptone, 0.5% meat extract, 0.1% Mg SO_4_.7H_2_O, and 0.5% K_2_HPO_4_. In the present investigation, MRS broth was used as basal medium because it is enriched with various carbon and nitrogen sources, enhancing the production of GPx and GST enzymes. Using the basal medium only, *L. plantarum* produced 147 U of GST per mg of protein and 28U of GPx per mg of protein after 24 h of incubation as clarified in the time-course of GST and GPx activities along with the biomass measurements of the test strain.

A preliminary single-factor tests were conducted to assess the impact of some production-stimulating parameters on the yield of GST and GPx produced by *L. plantarum* KU720558 when added to the basal medium. These experiments revealed that H_2_O_2_, SDS, urea, 1-butanol, Na Cl, amino acids, and pH adjustment stimulated GST production with little or no effect on the cell density, while bile salt, ethanol, and cooling at 4°C following incubation decreased the yield. For GPx activity, Na Cl, bile salt, H_2_O_2_, SDS, ethanol, and urea could improve the production without affecting the biomass, while amino acids, 1-butanol, aerobic incubation, and cooling at 4°C following incubation reduced the productivity (preliminary data were not shown). Milesi et al. (2008) and Pophaly et al. (2012) reported that *L. plantarum* isolates could respond to stressors through a complex network of reception and signaling. Additionally, they could tolerate harsh environmental conditions including acid, alkaline, detergent, osmotic, oxidative, and starvation. These conditions are usually associated with the production of stress-induced antioxidant and detoxification enzymes such as GPx and GST. Aguirre et al. (2006) mentioned that many fungal pathogens could fight against reactive oxygen species by H_2_O_2_ decomposing enzymes such as GPx as a stress-response mechanism. Huang et al. (2017) stated that mice treated with *L. plantarum* C88 following administration of aflatoxin B1 showed enhanced GPx and GST activities. Schwab et al. (2014) reported similar results. In this respect, *L. plantarum* KU720558 was subjected to a panel of stimulators to be investigated for the most significant parameters affecting the production of GST and GPx using the Placket-Burman statistical design. Later, each significant variable would be tested at three levels by the Box-Behnken design.

Concerning the Placket-Burman results for GST produced by *L. plantarum* KU720558, the most significant variables were H_2_O_2_, 1-butanol and amino acids. Li et al. (2007) and Li et al. (2010) stated that solventogenic *L. plantarum* could tolerate high concentrations of 1-butanol, up to 2.5% v/v, because the oxidative stress response was associated with regulation of the GST levels. In addition to conjugation capacities, bacterial GSTs could participate in the reduction of hydroperoxides to protect cells against products of oxidative metabolism (Fahey and Sundquist, 1991). Moreover, Kumagai et al. (1988) showed that the addition of L-cysteine and glycine to the fermentation medium induced GST activity in the yeast *Issatchenkia orientalis*. Vorobjeva et al. (1996) and Zhang et al. (2007) reported similar results. Placket-Burman data for GPx revealed top three significant factors including; sodium chloride, bile salt and urea. Li et al. (2003) and Dressaire et al. (2011) reported that GPx was overexpressed to protect the cells of the *Lactococcus lactis* subspecies *cremoris* SK11 from oxidative damage exerted by oxidizing agents, such as urea and hydrogen peroxide. This finding was explained by the study of Kim et al. (2006), who mentioned that GPx quenched the reactive oxygen species using its substrate (GSH) and hence increased the antioxidant ability of *L. brevis* KCTC 3498. Additionally, production of GPx by *Mucor hiemalis* was not affected by oxygen tension. This result seems to be contradictory because of the known protective properties of GPx against oxidative damage but is in agreement with the fact that lipoxygenase production, which is responsible for lipid peroxidation catalysis, by *Fusarium oxysporum* was enhanced in the presence of low oxygen tension (Aisaka et al., 1983).

Very little is known about the production of such enzymes from microbial sources through an optimized culture medium (Rao and Kavya, 2014). The addition of cumene hydroperoxide resulted in a 1.3-fold increase in the production of GPx (0.6 U/mg of protein) by *Mucor hiemalis*. Furthermore, the addition of increasing concentrations of H_2_O_2_ to the fermentation medium of *Saccharomyces cerevisiae* enhanced GPx productivity by 126%, reflecting that GPx activity was dependent on the presence of oxidative stress (Manfredini et al., 2004). *Bacillus* species produced 49 U of peroxidase after 18 h of incubation in an alkaline pH at 30°C. Furthermore, the addition of different amino acids, such as glycine, glutamine, L-cysteine, alanine, methionine, and asparagine, to the culture medium resulted in a pronounced production of peroxidase (50.7 U), particularly when asparagine was used (Rajkumar et al., 2013). In contrast, Rao and Kavya (2014) reported that *B. subtilis* produced 0.00045 units of peroxidase following optimization studies that involved pH and temperature adjustment (at 37°C and pH 6). For GST, Allocati et al. (2009) reported that the productivity of GST from *P. mirabilis* was increased by 1.3-folds in the presence of a nitrate source as well as by incubation under anaerobic conditions. Curti et al. (2014) stated that the addition of sorbitol improved the overall yield of GST produced by the yeast, *Pichia pastori*, by 3-folds using a medium enriched with yeast extract and casamino acids under the pH range 5–6. Additionally, the study of Skopelitou et al. (2012) reported that GST from *Agrobacterium tumefaciens* showed increased peroxidase activity (24.1 U) against the organic hydroperoxides and marked s-transferase activity against the aryl halides. Following optimization by Box-Behnken and analysis of our data, the optimized medium for GST consisted of MRS broth to which H_2_O_2_ (0.075%), SDS (0.05%), amino acids (0.0125%), 1-butanol (0.5%), and urea (0.1%) were added. The pH of the medium was adjusted to 6 and incubated for 24 h at 40°C under anaerobic conditions. For GPx, the optimum medium was composed of MRS broth supplemented with 0.17% urea, 0.025% bile salt, 7.5% Na Cl, 0.05% H_2_O_2_, 0.05% SDS, and 2% ethanol, at pH 6.0, and anaerobically incubated at 40°C for 24 h. Interestingly, growth under anaerobic conditions enhanced the activity of both enzymes. Similarly, Allocati et al. (2009) mentioned that GST productivity by *P. mirabilis* was enhanced under anaerobic incubation. However, several reports documented the effect of aerobic culture on the activity and expression of some antioxidants and ROS-scavenging enzymes in lactic acid bacteria such as catalase, superoxide dismutase, NADH oxidase/NADH peroxidase (NOX/NPR), and thioredoxin-thioredoxin reductase system. All of them activated to protect against O_2_ and related reactive species (Zotta et al., 2017; Maresca et al., 2018; Ricciardi et al., 2019).

Multiple isoforms of glutathione s-transferases were scarcely reported in bacterial cells in comparison to eukaryotic cells (Chee et al., 2014). In the present work, GPx and GST were purified using column chromatography resulting in 3- and 2-fold purified yield of 42.4 and 39.8%, respectively. Similar findings were reported by Yamashita et al. (2012), Chee et al. (2014), and Ibrahim et al. (2016). Furthermore, our purified GST and GPx showed a single protein band weighing 25 and 18 kDa, respectively, following SDS-PAGE and pIs of 7 and 5, respectively. However, Chee et al. (2014) mentioned that they purified and characterized two isoforms of glutathione s-transferase from *Acinetobacter calcoaceticus* Y1; GST1 and GST2. The first isoform showed a single protein band at 23 kDa following SDS-PAGE while GST2 formed some aggregates due to posttranslational modification presenting a molecular weight of 115 kDa. Furthermore, they calculated the pIs and found that GST1 had 4.5 while GST2 appeared at pI 6.2. Shi et al. (2014) reported that a GST protein with molecular weight 31 kDa was overexpressed in *E. coli*.

Regarding the effect of temperature on the enzymatic activities, our findings revealed general improvement in the enzymatic activities when incubated at 30 and 40°C. Although the later temperature degree was the optimal, the enzymes retained 70% of their activities at 50°C and lost about 36% of the activity at 60°C. Lower optimal temperatures for GST were reported in the literature for example; GST from Pseudomonas spp. DJ77 (30°C; Jung et al., 1996) and that of *E. coli* JM83 (35°C, Arca et al., 1999). On the reverse higher temperatures were also reported such as 50°C for GST from *Proteus mirabilis* (Federici et al., 2010). The impact of different pH values on the enzymatic activities was also assessed. Both enzymes were able to retain their activities at pH range 4–7 with optimal activity at pH 6. It was reported that GST from *Pseudomonas* spp. and *Pseudoalteromonas* spp. showed stability at pH range 6–9 while that purified from *Taenia solium* presented activity at acidic pH 4.5–8.5 (Jung et al., 1996; Plancarte et al., 2004; Wang et al., 2017). Additionally, the activities of our enzymes affected by different metal ions as well as some reagents. i.e., Fe^2+^ and DTT markedly reduced the GST relative activity by 60 and 68%, respectively. Moreover, Cu^2+^ showed the strongest decreasing effect (42.5%) on GPx relative activity. This might be due to the effect of DTT on the protein structure of the enzymes. Additionally, the metals might bind to the active site resulting in reduced activity (Salazar-Medina et al., 2010). Similar results were reported by Iizuka et al. (1989), Shi et al. (2014), and Wang et al. (2017).

Basically, the majority of cytosolic GSTs possess a molecular weight of 23–28 kDa. They are categorized into different classes including; alpha, mu, pi, theta, sigma, kappa, zeta, and omega depending on their primary structure and properties. Two binding sited are commonly present in most GSTs: G-site (for GSH attachment) and H-site (for substrate binding; Kim et al., 2009). In the present work, an amino acid sequences similarity check, done using BLASTp tool, confirmed that the two sequences were homologs of GST and GPx from *L. plantarum*. Additionally, both Query sequences of GST and GPx belonged to thioredoxin superfamily showing 73.3 and 76.6% identity, respectively. Therefore, sequences shared more than 40% similarity, belonged to the same class (Shehu et al., 2019). Furthermore, multiple sequence alignments with the top two protein sequences appeared in the hit list, revealed 20 conserved residues for GST C0HLL7. Shehu et al. (2019) reported that two structural motifs conserved in the majority of cytosolic GSTs, both bacterial and eukaryotic origins i.e., *N*-capping box (S/TXXD) as well as the hydrophobic staple motif, which are essential for the enzymatic folding and stability. The *N*-capping box, characterized by a reciprocal backbone side-chain hydrogen bond interaction between the *N*-cap (Ser/Thr) and N3 (Glu/Asp) regions, is involved in the formation and stability of α-helices. If Glycine was present, four amino acids before the *N*-cap residue is considered conserved. In the current work, GST showed similar findings where the *N*-capping box was consisted of Thr27 while the N3 region showed conserved Asp30. Interestingly, glycine residue was part of a buried local sequence: **G**-X-X-**h**-**T**-X-X-**D** that was conserved in all cytosolic GSTs from mammals to bacteria (Shehu et al., 2019). Moreover, these characters could classy the cytosolic GST C0HLL7 to beta class, which is present only in bacteria. It was also able to conjugate CDNB and was also purified by GSH-affinity chromatography as stated also by Shehu et al. (2019). For GPx, Tosatto et al. (2008) reported the presence of four important conservation components of GPx active site including; S, Gln, Trp, and Asn. Moreover, mutational substitution of Asn by His, Ala, or Asp and also replacement of Trp by f might occur. Additionally, these conserved residues were present inside a core that extended from the catalytic site to the opposite protein surface. Beside the above residues, there were other hydrophobic ones located within the core. These findings were in consistent with our results.

The 3D model of GST C0HLL7 sequence showed that the percent identity and similarity between the target sequence and selected template are 34 and 46%, respectively with high confidence. Shehu et al. (2019) reported that the overall crystal structures of GST classes composed of an *N*-terminal domain similar to the thioredoxin-like protein fold. Additionally, helical *C*-terminal domain was detected, which was separated by a short linker. Furthermore, a unique structural motif, detected only in beta class GST, was present at the G-site and it composed of a network of hydrogen bonding needed to zipper the *C*-terminal domain end and hence starting helix of the thioredoxin-like domain and these findings were in consistent with our data. For GPx C0LL8 sequence, it showed percent identity between the template and the query is 35% and the similar amino acids are 50% of the sequence. The generated model showed two helical structures, two strands and loops. Possible protein cavities were explored to define druggable sites. GST model showed two considerably large pockets, 185 Å3 and 71 Å3, with druggability score in the range of 0.5-0.8. The two cavities are connected and can form a large pocket. While in case of the peroxidase model, it has two pockets as well with relatively smaller volumes, 71 Å3 and 32 Å3. The two pockets are connected and show druggability scores of 0.65 and 0.66. Tripathi and Kellogg (2010) mentioned that protein pockets and cavities aid in prediction of the binding sites. The two models were also mapped to identify the electrostatic properties of the surface patches. Upon analyzing the structures of both enzymes, some thermal stability (up to 60°C and little at 70°C) appears to be due to a significant amount of hydrophobic regions on the protein surface while polar patches were smaller. Ferrandi et al. (2018) reported that hydrophobic interactions considered an important feature of a protein to identify its thermostability.

## Conclusion

In conclusion, Placket-Burman and Box- Behnken statistical designs were used for evaluating the effective parameters and their optimal concentrations to enhance GST and GPx enzyme productivity by *L. plantarum* KU720558. They allowed the development of empirical polynomial models for the production of GST and GPx by the test strain. The most important variables affecting the production of the GST enzyme were amino acids, hydrogen peroxide and 1-butanol, while those involved in the production of GPx were bile salt, sodium chloride, and urea. The optimal conditions for GST production involved the basal medium supplemented with 0.1% urea, 0.075 H_2_O_2_, 0.5% 1-butanol, 0.0125% amino acids, and 0.05% SDS at pH 6.0 and anaerobically incubated for 24 h at 40°C. Furthermore, the conditions used for enhancement of GPx production included 0.17% urea, 0.025% bile salt, 7.5% Na Cl, 0.05% H_2_O_2_, 0.05% SDS, and 2% ethanol added to MRS broth at pH 6.0 and anaerobically incubated for 24 h at 40°C. Optimization led to a 12- and 22-fold increase in the productivity of GST and GPx enzymes, respectively, compared to the basal medium, recommending their application in the industry. Furthermore, the properties of the purified GST and GPx showed maximum catalytic activities at 40°C and pH 6. There was a detailed structural analysis for both enzymes leading to proper understanding of their stability and activity.

## Data Availability Statement

The raw data supporting the conclusions of this article will be made available by the authors, without undue reservation, to any qualified researcher.

## Author Contributions

LA-M conceived the experiments. LA-M, SA, AR, and EK conducted the experiments. SA, LA-M, EF, and EK analyzed the results. KE was responsible for the structure elucidation and modeling. All authors participated in the manuscript revision.

### Conflict of Interest

The authors declare that the research was conducted in the absence of any commercial or financial relationships that could be construed as a potential conflict of interest.
