# Supplementary material for: Stress-Based Production, and Characterization of Glutathione Peroxidase and Glutathione S-Transferase Enzymes From Lactobacillus plantarum
Source: Front Bioeng Biotechnol. 2020 Feb 27;8:78. doi: 10.3389/fbioe.2020.00078 (PMC7057912; doi:10.3389/fbioe.2020.00078)
Supplement: Supplementary file 1 [file Table_1.DOCX]

**Table S1: Concentrations of the variables used in Box-Behnken design matrices of s-transferase and peroxidase activities.**

|  |  | **Concentrations of variables in percentage (%)** | | | |  |
| --- | --- | --- | --- | --- | --- | --- |
|  | **variables (-1) (0)** | |  |  | **(1)** |  |
|  | **Urea** 0.05 0.1 | |  |  | 0.15 |  |
|  | **Bile salt** 0.025 0.05 | |  |  | 0.075 |  |
|  | **H_2_O_2_** 0.025 0.05 | |  |  | 0.075 |  |
|  | **Na Cl**  2.5 5 | |  |  | 7.5 |  |
|  | **1-Butanol** 0.5 1  **Amino acids** 0.0125 0.025 | |  |  | 1.5  0.05 |  |

**Table S2:** Regression coefficients of GST and GPx activities (Placket-Burman design).

|  | **s-transferase** | | | **peroxidase** | | ***P-value*** |
| --- | --- | --- | --- | --- | --- | --- |
| **Variables** | ***Coefficients*** | ***t Stat*** | ***P-value*** | ***Coefficients*** | ***t Stat*** |  |
| **intercept** | 314.4375 | 5.299075 | 0.033816 | 77.1875 | 4.931711 | 0.038742 |
| **Na Cl (X1)** | -52.7644 | -0.66254 | 0.575764 | 50.34368 | 2.396616 | 0.013876 |
| **Bile salt (X2)** | 20.27719 | 0.249492 | 0.826266 | 53.22154 | 2.482678 | 0.013108 |
| **H_2_O_2_ (X3)** | 87.15024 | 1.205967 | 0.035113 | 10.26435 | 0.538497 | 0.64415 |
| **SDS (X4)** | 24.46707 | 0.334216 | 0.770009 | 32.94899 | 1.706368 | 0.230059 |
| **Ethanol (X5)** | 33.64356 | 0.420165 | 0.715202 | 49.60507 | 2.34871 | 0.143311 |
| **Urea (X6)** | 6.3839 | 0.079651 | 0.943767 | 58.08034 | 2.74739 | 0.011087 |
| **1-butanol (X7)** | 125.5463 | 1.599141 | 0.025090 | -14.0308 | -0.67756 | 0.567922 |
| **Aerobic incubation (X8)** | -31.1849 | -0.39722 | 0.729589 | -80.8102 | -3.90242 | 0.598322 |
| **Amino acids (X9)** | 88.84293 | 1.108483 | 0.038310 | -53.3405 | -2.52318 | 0.127676 |
| **pH (X10)** | -80.4821 | -1.00512 | 0.420684 | -35.9741 | -1.70331 | 0.230623 |
| **Temperature (X11)** | 84.41432 | 1.153087 | 0.368075 | 24.59882 | 1.273928 | 0.330706 |
| **Incubation time (X12)** | 10.78323 | 0.149216 | 0.895071 | 16.96637 | 0.890104 | 0.467327 |
| **Cooling (X13)** | -181.171 | -2.22914 | 0.155596 | -56.7549 | -2.6475 | 0.117953 |

**Table S3:** Regression coefficients of GST and GPx activities (Box-Benkhen design).

|  | **s-transferase** | | | **peroxidase** | | ***P-value*** |
| --- | --- | --- | --- | --- | --- | --- |
| **Variables** | ***Coefficients*** | ***t Stat*** | ***P-value*** | ***Coefficients*** | ***t Stat*** |  |
| Intercept | 890 | 12.00567 | 0.001243 | 182 | 1.343442 | 0.027171 |
| X1 | -354 | -13.5066 | 0.000878 | 69.25 | 1.445813 | 0.024400 |
| X2 | 7.25 | 0.276617 | 0.800037 | -67.125 | -1.40145 | 0.025561 |
| X3 | 103.5 | 3.948951 | 0.028964 | 99.375 | 2.074768 | 0.012964 |
| X_1_^2^ | 274.25 | 5.593118 | 0.011289 | -139.625 | -1.55819 | 0.021707 |
| X_2_^2^ | -14.25 | -0.29062 | 0.790276 | 42.625 | 0.475689 | 0.066678 |
| X_3_ | 68.75 | 1.402103 | 0.255442 | 177.625 | 1.982269 | 0.014174 |
| X1X2 | -65 | -1.75364 | 0.177772 | -5.5 | -0.0812 | 0.094039 |
| X1X3 | -112 | -3.02165 | 0.056685 | 121.5 | 1.793716 | 0.017075 |
| X2X3 | -347 | -9.36172 | 0.002581 | 22.75 | 0.33586 | 0.075909 |


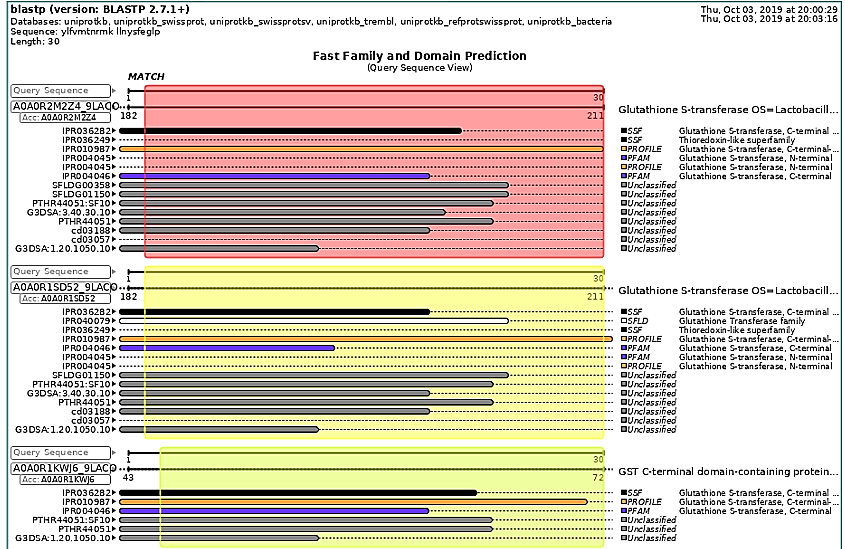


(A)

(B)


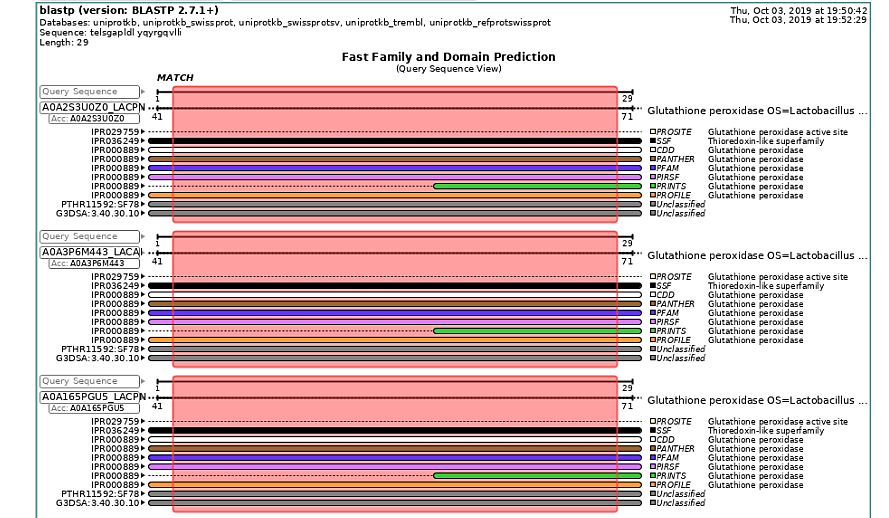


**Figure (S4):** Similarity between (A) Query GST (C0HLL7) or (B) Query GPx (C0HLL8) with different *L. plantarum* enzymes using BLASTp/functional prediction tool, showing the related family (thioredoxin-like superfamily). <https://www.ebi.ac.uk/Tools/services/web/toolresult.ebi?jobId=ncbiblast-I20191003-195040-0895-61381666-p2m&analysis=ffdp-query>
